# Case Report: Diffuse idiopathic skeletal hyperostosis with ossification of the posterior longitudinal ligament in the cervical spine: A rare case with dysphagia and neurological deficit and literature review

**DOI:** 10.3389/fsurg.2022.963399

**Published:** 2022-08-09

**Authors:** Chaoyuan Li, Wenqi Luo, Hongchao Zhang, Jianhui Zhao, Rui Gu

**Affiliations:** Departments of Orthopedics Surgery, China-Japan Union Hospital, Jilin University, Changchun, China

**Keywords:** diffuse idiopathic skeletal hyperostosis, cervical spine, literature review, OPLL, dysphagia

## Abstract

Diffuse idiopathic skeletal hyperostosis (DISH) is characterized by the calcification and ossification of ligaments and tendons. Progressive dysphagia caused by DISH-related anterior cervical osteophytes and deteriorating dysphagia caused by DISH combined with neurological dysfunction resulting from the posterior longitudinal ligament is rare. The initial diagnosis is misleading and patients often consult several specialists before spine surgeons. This study aims to provide a comprehensive review of the literature on this challenging pathological association. We also present a case illustration where a 53-year-old man presented with progressive dysphagia and foreign body sensation in the pharynx, accompanied by a neurological numbness defect in the right upper limb. Radiography and computed tomography confirmed the existence of osteophytes at the anterior edge of the C4–C7 pyramid and ossification of the posterior longitudinal ligament, in which the giant coracoid osteophyte could be seen at the anterior edge of the C4–C5 pyramid. The anterior cervical osteophyte was removed, and decompression and fusion were performed. The symptoms were relieved postoperatively. No recurrence of symptoms was found during the six-month follow-up. Spine surgeons should consider progressive dysphagia caused by DISH-related osteophytes at the anterior edge of the cervical spine as it is easily misdiagnosed and often missed on the first evaluation. When combined with ossification of the posterior longitudinal ligament, following cervical osteophyte resection it is necessary to consider stabilizing the corresponding segments *via* fusion.

## Introduction

Hyperosteogeny at the anterior edge of the cervical spine mainly results from cervical degeneration. It has been reported in other diseases such as diffuse idiopathic skeletal hyperostosis (DISH), ankylosing spondylitis, acromegaly, hypoparathyroidism, and trauma, of which DISH and ankylosing spondylitis are the most common (1). Osteophytes at the anterior edge of the cervical spine can cause a series of clinical symptoms, such as progressive dysphagia, foreign body sensation, pain during swallowing (2), cervical stiffness and pain, and dyspnea and dysphonia (3–5), among others. In a study by Strasser et al. (6), only 1.7% of patients with osteophytes had dysphagia. When dysphagia occurs, the C5–C6 vertebrae are most often involved, followed by C4–C5, C2–C3, and C3–C4 (7). Surgical resection of hyperplastic osteophytes is an effective method for treating severe dysphagia (8–10). However, the clinical characteristics of patients with DISH-related anterior cervical osteophyte hyperplasia with ossification of the posterior longitudinal ligament (OPLL) have only been sporadically reported in the literature, and the best treatment has not been elucidated. Patients with posterior longitudinal ligament ossification (OPLL) may have varying degrees of neurological symptoms, including radiculopathy and myelopathy. The prevalence of OPLL in Japan and East Asian countries ranges from 1.9 to 4.3% (11). Herein, we report a rare case of DISH complicated by OPLL, with progressive dysphagia and neurological dysfunction and provide a comprehensive review of the literature on this challenging pathological association.

## Methods

According to the PRISMA (Preferred Reporting Items for Systemic Reviews and Meta-Analyses) statement, we systematically reviewed literature about dysphagia caused by DISH-related cervical osteophyte hyperplasia, which were published before May 2022. And on the basis of literature review, we provide a case report. PubMed, EMBASE, Web of Science and the Cochrane Database of Systemic Reviews were searched using these keywords: (“diffuse idiopathic skeletal hyperostosis” or “Forestier” or “Forestier syndrome” or “forestier's disease”) and (“dysphagia” or “deglutition disorder”). Keywords referred to medical subject heading (MeSH). The search terms used on PubMed were: ((“Deglutition Disorders"[Mesh]) OR (((((((((Deglutition Disorder[Title/Abstract]) OR (Disorders, Deglutition[Title/Abstract])) OR (Swallowing Disorders[Title/Abstract])) OR (Swallowing Disorder[Title/Abstract])) OR (Dysphagia[Title/Abstract])) OR (Oropharyngeal Dysphagia[Title/Abstract])) OR (Dysphagia, Oropharyngeal[Title/Abstract])) OR (Esophageal Dysphagia[Title/Abstract])) OR (Dysphagia, Esophageal[Title/Abstract]) AND (meta-analysis[Filter]))) AND ((“Hyperostosis, Diffuse Idiopathic Skeletal"[Mesh]) OR ((((((((((((((((Diffuse Idiopathic Skeletal Hyperostosis[Title/Abstract]) OR (Vertebral Ankylosing Hyperostosis[Title/Abstract])) OR (Ankylosing Hyperostoses, Vertebral[Title/Abstract])) OR (Ankylosing Hyperostosis, Vertebral[Title/Abstract])) OR (Hyperostoses, Vertebral Ankylosing[Title/Abstract])) OR (Hyperostosis, Vertebral Ankylosing[Title/Abstract])) OR (Vertebral Ankylosing Hyperostoses[Title/Abstract])) OR (Forestier's Disease[Title/Abstract])) OR (Disease, Forestier's[Title/Abstract])) OR (Forestiers Disease[Title/Abstract])) OR (Forestier-Rotes Disease[Title/Abstract])) OR (Disease, Forestier-Rotes[Title/Abstract])) OR (Forestier Rotes Disease[Title/Abstract])) OR (Ankylosing Vertebral Hyperostosis with Tylosis[Title/Abstract])) OR (Forestier Disease[Title/Abstract])) OR (Disease, Forestier[Title/Abstract]))).

For inclusion in this literature review, they had to meet all of the following criteria: (1) the study participants were over 18 years old; (2) they were clearly diagnosed with DISH which led to dysphagia; (3) there was no history of cervical surgery; (4) an adequate clinical and/or radiologic description of each individual case, and their medical history was relatively complete. (5) the study was a case report, case series or observational study. Studies were excluded for any of the following: (1) the study was a review, meeting abstract, non-clinical study, or *in vitro* study; (2) inferior quality literature or with insufficient outcome indicators; (3) lesions were not in the cervical spine or associated with trauma. (4) non-English publications. All selected studies were independently reviewed by 2 investigators for inclusion in the final analysis. Any inconsistencies were resolved by discussion until a consensus was reached.

## Results

**Figure 1:**
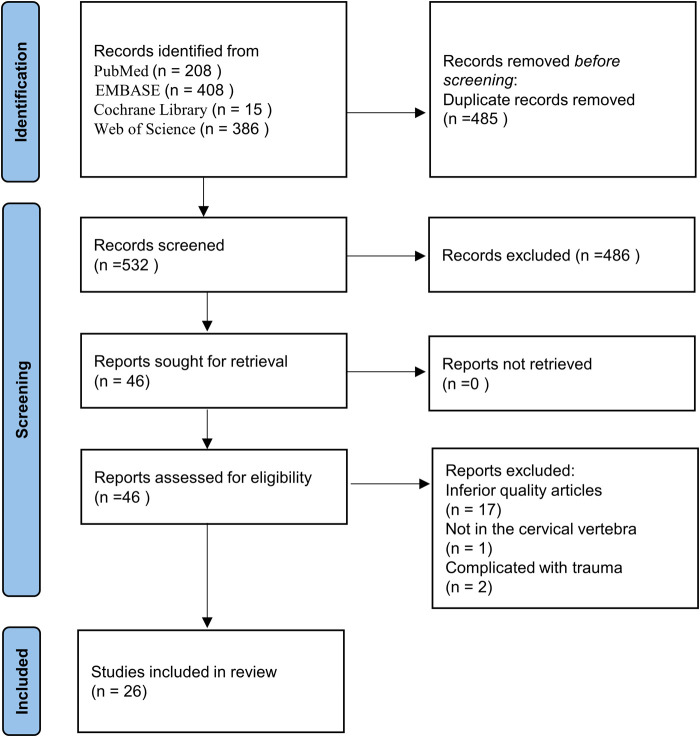
Flow chart showing the process of article selection.

A total of 1017 relevant articles from August 1963 to May 2022 were initially identified from PubMed (*n* = 208), EMBASE (*n* = 408), Cochrane Library (*n* = 15), and Web of Science (*n* = 386). After exclusion of duplicates, 532 articles remained. According to the inclusion and exclusion criteria, 486 records considered irrelevant by title or abstract were excluded. Subsequently, the full texts of the remaining 46 articles were checked, and 20 articles were excluded with reasons: inferior quality articles (*n* = 17); not in the cervical vertebra (*n* = 1); complicated with trauma (*n* = 2). Finally, the study included 26 articles involving 124 cases (Figure 1).

In the 124 cases reported in the literature (Table 1), the anatomical plane of the osteophytes was identified in 72 cases. The osteophytes were at the junction of the fourth and fifth cervical vertebrae in 55 cases (76%), at the junction of the fifth and sixth cervical vertebrae in 41 cases (57%), at the junction of the third and fourth cervical vertebrae in 38 cases (53%), at the junction of the sixth and seventh cervical vertebrae in 25 cases (35%), and at the junction of the second and third cervical vertebrae in 12 cases (17%). Of the 124 cases reported in the literature (Table 2), dysphagia was the most common complaint; some patients also complained of swallowing pain, foreign body sensation, neck pain, spinal stiffness and dysphonia. Among them, 5 patients lost a significant amount of weight, and some patients had a history of esophageal reflux and aspiration pneumonia.

**Table 1:** Literature review of anterior osteophytes of the cervical spine leading to dysphagia.

Ref No.	Author	No. Patients	Combined OPLL and DISH	Male/Female	Country	Age years (Median)	Follow-up (mo)	Recurrence
(12)	Murayama et al.	1	Yes	1/0	Japan	70	12	0
(13)	Giammalva et al.	1	Yes	1/0	Italy	65	NP	NP
(14)	Anshori et al.	1	Yes	0/1	Indonesia	59	NP	0
(1)	De Jesus-Monge et al.	1	Yes	1/0	Puerto Rico	80	NP	(died)
(8)	Miyamoto et al.	7	1 of them	6/1	Japan	65	108	2
(15)	Yoshioka et al.	4	Yes	4/0	Japan	67.3	NP	NP
(16)	Castellano et al.	5	Yes	4/1	USA	75.4	4.8	0
(9)	Urrutia et al.	5	No	5/0	Chile	71	59.8	0
(17)	von der Hoeh et al.	6	No	6/0	Germany	67	23	0
(4)	Caminos et al.	1	No	1/0	Spain	75	NP	NP
(18)	Presutti et al.	12	No	11/1	Italy	65	26	0
(19)	Egerter et al.	2	No	2/0	USA	65.5	24.5	0
(20)	Mattioli et al.	21	No	18/3	Italy	70.6	66	1
(21)	Lui Jonathan et al.	6	No	6/0	United Kingdom	59	42.3	0
(22)	McCafferty et al.	7	No	7/0	USA	70.6	11	0
(23)	Scholz et al.	5	No	5/0	Germany	61.6	70	1
(24)	Suzuki et al.	2	No	1/1	Japan	58	42	2
(10)	Oppenlander et al.	5	No	5/0	USA	67.8	4.6	0
(25)	Kawamura et al.	5	No	4/1	Japan	75	NP	NP
(26)	Kos et al.	2	No	2/0	The Netherlands	70	2.75	0
(27)	Lecerf and Malard	2	No	2/0	France	79.5	NP	NP
(28)	Laus et al.	6	No	6/0	Italy	60	16	0
(29)	Montinaro et al.	3	No	3/0	Italy	59.7	NP	NP
(30)	Ido et al.	3	No	3/0	Japan	69.7	46.7	0
(31)	Carlson et al.	6	No	5/1	USA	73	NP	NP
(32)	Nelson et al.	5	No	4/1	USA	78.8	12	0

Abbreviations: No. patients, number of patients; NP, not precised.

**Table 2:** Summary of clinical characteristics, radiology, treatment, and outcome characteristics.

Localization	Other symptoms	Risk Factors	Treatment (outcome)	Additional Information
C4–C5	odynophagia	Diabetes	Conservative treatment: non-steroidal anti-inflammatory drugs, corticosteroids, and muscle relaxants (Easy to relapse)	The hospital stay is 4–11 days.
C5–C6	foreign-body sensation	Hypertension	Osteophyte excision (NP)	
C3–C4	spinal rigidity or neck pain	Smoking	Additional fusion procedures (NP)	
C6–C7	cough			
C2–C3	neurological deficit			
	dyspnea and dysphonia			

**Figure 2:**
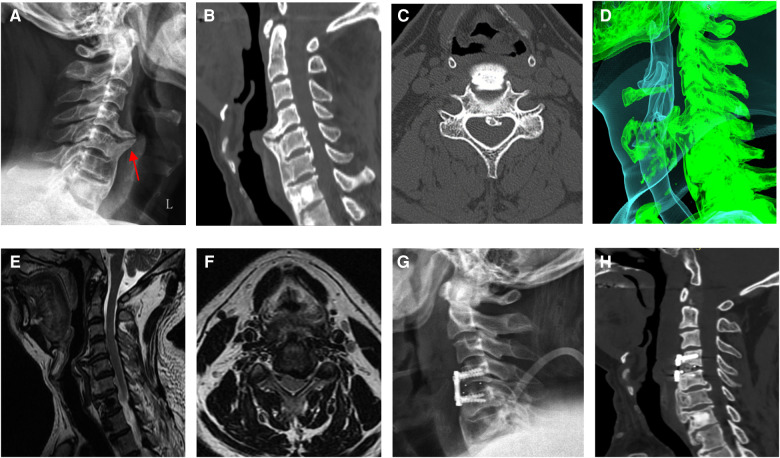
(**A**) osteophyte formation can be seen in front of the C4–C7 vertebral body on cervical radiographs before operation, and a giant coracoid osteophyte can be seen at the anterior edge of C4–C5. (**B,C**) The C4–C5 segment showed the formation of a huge osteophyte in the anterior margin and ossification of the posterior longitudinal ligament in the spinal canal. (**D**) Preoperative three-dimensional computed tomography (CT) reconstruction showing that hyperplastic osteophytes compressed the trachea and esophagus. (**E,F**) Preoperative magnetic resonance imaging showing that the spinal canal space became narrower at the C4–C5 level. (**G,H**) Radiographic and CT examination after operation showed that the large osteophyte in front of the C4-C5 vertebral body had been removed.

**Figure 3:**
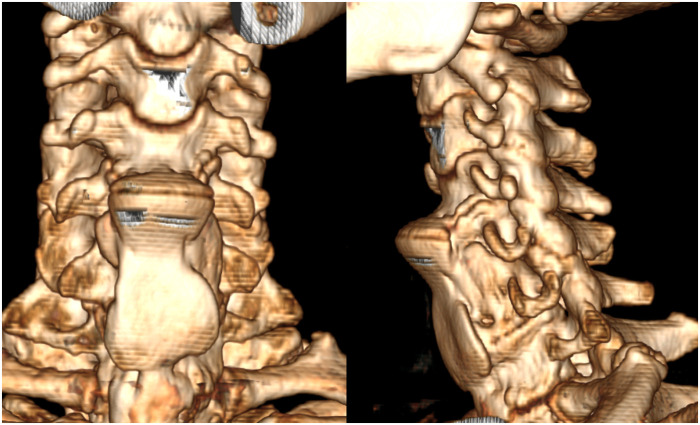
The shape of the osteophyte can be clearly seen with 3D reconstructions of cervical vertebrae with CT without soft tissue window.

As described above, the patient in our case report was admitted to the hospital with the typical complaint of progressive dysphagia. The patient aged 53 had persisted for eight months and was aggravated for five months and diagnosed with DISH complicated by OPLL, with rare symptoms of dysphagia and neurological deficits. Prior to admission, the patient had been treated in several hospitals; however, physicians failed to make an accurate diagnosis, and the patient's symptoms continued to worsen. Physical examination showed numbness in the right upper limb, muscle strength was not obviously abnormal, and the Spurling test was positive. He had a history of diabetes for 19 years and his fasting blood glucose was high at 8.90 mmol/L. Cervical radiography showed osteophytes in front of the C4–C7 vertebral body, and giant coracoid osteophytes at the anterior edge of the C4–C5 cone (Figure 2A). Cervical computed tomography (CT) showed hyperosteogeny at the anterior margin of the cervical vertebra. A large osteophyte could be seen in front of the C4–C5 vertebral body pressing forward against the esophagus, and OPLL could be seen at the back (Figures 2B–D). The shape of the osteophyte could be clearly seen with 3D reconstructions of cervical vertebrae with CT without a soft tissue window (Figure. 3). Preoperative cervical magnetic resonance imaging (MRI) showed that the anterior edge of the dura mater of the C4–C5 segment was compressed, the spinal canal space was narrowed, and the esophagus was displaced (Figures. 2E,F). Four days after admission, the diagnosis was clear and the patient underwent fusion surgery considering the instability of stage C4–5 and mild neurological symptoms. Large beak-like osteophytes were removed using an anterior cervical approach. At the same time, the C4–C5 segmental discs were removed and fused. After the operation, the patient reported an improvement in dysphagia symptoms as well as the numbness in the upper right limb. Radiographic and CT re-examination showed that the larger osteophyte in front of the C4–C5 vertebral body had been successfully removed (Figures 2G,H) and the patient was administered non-steroidal anti-inflammatory drugs after surgery. Three days after the operation, the patient was discharged from the hospital. No recurrence of symptoms was found at the six-month follow-up.

## Discussion

Dysphagia caused by a large cervical osteophyte is rare. The incidence of asymptomatic hyperosteogeny at the anterior edge of the cervical vertebra is 20%–30% (33) and is more common in middle-aged and elderly men over 50 years of age (34). Although the incidence of this disease is high, most do not show clinical symptoms; however, large osteophytes may compress the pharynx wall or esophagus, resulting in dysphagia. Strasser et al. studied 3,318 patients with osteophytes at the anterior edge of the cervical vertebrae and found that 55 patients had dysphagia, with an incidence of approximately 1.7% (6). Cervical osteophytes can cause dysphagia through paraesophageal tissue inflammation or mechanical compression of the pharynx or esophagus (35). In addition, previous findings suggest that the degree of dysphagia is significantly correlated to the location, size, and number of osteophytes (36). The symptoms caused by osteophytes differ in different segments (37). When osteophytes appeared in C2–C4, there was not only dysphagia but also tracheal obstruction symptoms, such as dyspnea and sleep apnea. When the osteophyte appeared in C4–C7, dysphagia was the main symptom. In this case, the C4–C7 segment osteophyte was formed and fused, and a large beak osteophyte was formed in the C4–C5 segment, which caused dysphagia.

DISH is one of the most common causes of dysphagia caused by anterior cervical hyperosteogeny. The main diagnostic criteria for DISH include anterolateral ossification of at least four adjacent vertebrae, relative constant disc height, no facet joint stiffness, or sacroiliac joint sclerosis (10). The C4–C7 vertebral bodies were fused by the anterior bony spur in this case, while the intervertebral disc height was relatively preserved; therefore, the patient was diagnosed with DISH. There was also no ankylosis of the facet joints or sclerosis of the sacroiliac joint. Although the incidence of dysphagia caused by huge cervical osteophytes is low, there have been cases of discomfort while swallowing or a foreign body sensation, and organic lesions of the esophagus and its surrounding tissues, such as esophagitis and tumors, can also cause such symptoms. Therefore, radiography, CT, MRI, and endoscopy are needed simultaneously to confirm the diagnosis.

DISH tends to occur in older men with metabolic syndrome, diabetes, or obesity. Growth hormones and insulin-like growth factors promote bone growth (38). In our case, the patient had a 19-year history of diabetes, which may have been the cause of the multiple ossifications. The Japanese Investigation Committee for the Ossification of the Spinal Ligaments (39) identified four OPLL subtypes according to the severity of the disease: (1) localized, limited to intervertebral disc space; (2) segmental, located behind each vertebral body, no adjoined adjacent vertebral segments; (3) continuous, crossing the intervertebral disc between several segments; and (4) mixed, segmental and continuous. In our case, the patient was diagnosed with the segmental type.

Early lesions can be conservatively treated through improved diet and use of non-steroidal anti-inflammatory drugs, corticosteroids, and muscle relaxants (40). Surgical treatment is often required for patients in whom conservative treatment fails, or if the symptoms seriously affect their diet. Surgical treatment includes direct anterior resection of the osteophytes and preservation of the anterior ring (17). This type of osteophyte resection usually relieves symptoms, avoids some complications of bone plate fixation, and greatly reduces the financial burden on the patients. Miyamoto et al. (8) compared the preoperative, postoperative, and long-term dysphagia and imaging results of seven patients who underwent anterior cervical osteophyte resection. The average growth rate of postoperative osteophytes was approximately 1 mm/year, and the recurrence rate of high-activity segments was significantly higher than that of low-activity segments. Some scholars believe that DISH is mainly due to cervical degeneration, intervertebral instability, anterior edge osteophyte hyperplasia, and compression of the esophagus (41). In theory, internal fixation of the bone plate is required after osteophyte resection to achieve intervertebral stability. However, the titanium plate itself has a certain thickness, which leads to esophageal compression again after the operation. This results in the symptoms of dysphagia not being relieved or not relieved completely, and complications, such as loosening and failure, may occur. In addition, inspired by measures to prevent ossification after hip arthroplasty, oral non-steroidal anti-inflammatory drugs (such as indomethacin) and short-term radiotherapy after surgery are worthy of in-depth study (41). In this case, the lesion segment of the patient was considered to be the C4–5 segment with a high range of motion; therefore, anterior cervical osteophyte resection and internal fixation fusion were adopted, and nonsteroidal anti-inflammatory drugs were administered orally after the operation.

Our patient presented with progressive dysphagia and numbness of the right upper limb. CT revealed huge osteophytes in the C4–C5 segment, oppressing the pharynx, and OPLL pressing on the cervical spinal cord. Surgical treatment was a reasonable treatment option. In a similar case, Murayama et al. (12) reported a 70-year-old male patient with severe dysphagia and mild myelopathy treated with anterior vertebral resection, interbody fusion, posterior decompression, and posterior-stabilized lateral mass screws. However, in another similar case, the patient was treated conservatively because of cervical DISH with OPLL but normal neurological function (14). When DISH is complicated by OPLL, to determine the most appropriate treatment several conditions must be considered. When the neurological signs or symptoms are mild and there is no evidence of myelopathy, conservative treatment should be considered (42). As long-term spinal cord compression may lead to irreversible damage, surgical decompression is recommended through cervical OPLL when myelopathy is obvious. However, the best surgical approach is still controversial. Anterior vertebral resection and fusion after ossification is a radical surgical method, which is most suitable for local or segmental OPLL in patients without congenital stenosis, involving less than three vertebrae (42, 43). In contrast, the posterior approach (42), mainly laminoplasty, is widely used to treat high-risk patients over 65 years of age with multi-segmental diseases and non-kyphosis. In the present case, the patient had severe dysphagia and mild nerve root symptoms. The neurological symptoms mainly originated from the C4–5 segment, and there was no myelopathy. After a comprehensive consideration, osteophyte removal, anterior decompression, and fusion were performed. Postoperative dysphagia gradually improved. Neurological symptoms also improved by stabilizing the OPLL. During the follow-up, we used the eating assessment tool (EAT-10) questionnaire to subjectively evaluate the patient; his score was 1. The result of EAT-10 was considered indicative of abnormal swallowing if the score was 3 or more (44). The patient did not complain of further dysphagia.

## Conclusion

Progressive dysphagia caused by an anterior cervical osteophyte is very rare and easily misdiagnosed. For patients with progressive dysphagia, the possibility of hyperosteogeny at the anterior edge of the cervical spine should be considered in differential diagnosis. After a definite diagnosis is made, a surgical strategy must be considered. If present, OPLL should be carefully considered when selecting the method of surgery. Anterior decompression and fusion after osteophyte resection can effectively prevent the instability of osteophyte resection segments and osteophyte regeneration, and, in stabilizing the OPLL, can relieve neurological symptoms.

## Data Availability

The raw data supporting the conclusions of this article will be made available by the authors, without undue reservation.
